# A comparative study: quality of life, self-competence, and self-liking among the caregivers of children with attention deficit hyperactivity disorder and other non-ADHD children

**DOI:** 10.1186/s43045-022-00189-x

**Published:** 2022-03-30

**Authors:** Mona Gamal Abd Elnaser Ahmed, Ebaa Marwan Felemban, Marwa Abd El-fatah Ali El-slamoni

**Affiliations:** 1 Psychiatric and Mental Health Nursing, Faculty of Nursing, Zagazig University, Zagazig, Egypt; 2 Department of Nursing, College of Applied & Medical Science, Taif University, Taif, Kingdom of Saudi Arabia

**Keywords:** Quality of life, Self-competence, Self-liking, Caregivers, ADHD

## Abstract

**Background:**

The presence of attention deficit hyperactive disorder (ADHD) causes great impairment in social and professional activities as well as in the quality of life (QOL) and increases level of parental stress.

**Objective:**

This study aimed to compare the QOL, self-competence, and self-liking among the caregivers of ADHD and non-ADHD children.

**Method:**

A descriptive cross-sectional study was conducted on 216 caregivers. They were divided into two groups: caregivers of children with ADHD and caregivers of children without ADHD.

**Setting:**

This study was conducted in the Elsinbellawin City at Dakahlia Governorate in Egypt. Tools used were socio-demographic data sheet and WHO Quality of Life Scale-Brief (WHOQOL-Brief) and self-liking/self-competence scale.

**Results:**

Our results showed that all QOL dimensions of the caregivers of ADHD children were markedly deteriorated, if compared to those of non-ADHD children, and two thirds of caregivers of ADHD children suffered from poor level quality of life (QOL). Caregivers of non-ADHD children had fair level of self-competence but a good level of self-liking. Caregiver of ADHD children who had no work or history of medical diseases has a fair level of QOL and self-competence but a good level of self-liking.

**Conclusions:**

We found that caregivers of ADHD children have fair levels of QOL, self-liking, and self-competence, if compared to those of non-ADHD children. There are statistically significant correlations between QOL, self-competence, and self-liking among both caregivers of both non-ADHD children and ADHD children.

## Background

According to the American Psychiatric Association, attention deficit hyperactivity disorder (ADHD) is a complex neurodevelopmental disorder characterized by a cluster of symptoms of inattention and disorganization, with or without hyperactivity-impulsivity [1]. ADHD is one of the most pervasive childhood psychiatric disorders, affecting 3–7% of school-age children worldwide, in which genetic and environmental factors play crucial roles. If no proper treatment, children with ADHD may suffer from academic underachievement, emotional regulation impairment, physical injuries, and unsatisfactory relationships with family, teachers, and peers or even comorbid behaviors and/or mood disorders [2]. The ADHD effects are not confined to the child suffering alone but also have impact on his family, school, and other direct social environments, as having a child with ADHD increases family stress, emotional distress, exhaustion, and problems, while reducing their efficacy. Parents of ADHD children have higher guilt because their perceptions of their role demands exceed their resources to cope with them. Their life is concentrated on the symptoms, leaving inadequate time and energy for meeting the developmental needs of the ADHD child as well as other family members, increasing the susceptibility to depression and having a lower quality of life (QOL) [3].

QOL is defined as the expected well-being and satisfaction of one’s life achievements in the social and culture contexts. It is a subjective and multidimensional construct consisting of physical, emotional, and social domains including both cognitive (satisfaction) and an emotional (happiness) component [4]. Evidence indicates QOL is not significantly influenced with ADHD symptoms or intellectual abilities alone but with his internalizing/externalizing problems, social skills, or adaptive behavior. Although QoL is influenced by many proximal (e.g., family and friendship) and distal (e.g., socio-economic and cultural) factors, the presence of ADHD has been considered as one of the most potent risk factors for worse QoL scores [5].

A lower QOL was associated with the child’s functioning impairment, and the few social support received the use of non-adaptive coping mechanisms, and emotional conduct, physical health, and coordination problems as well as maternal and paternal mental health problems. Parents of children with ADHD reported more family problems and are at a higher risk for developing physical or psychological problems [6].

Self-esteem is composed of both self-liking and self-competence. Self-competence refers to seeing oneself qualified, successful, and skillful in achieving goals. It is important for the development of the personality and for learning in general via its ability to motivate oneself, cope with failure, and handle one’s emotions. Self-liking refers to self-worth and value due to an individual’s place estimation in the social setting, irrespective of their success, reflecting his self-acceptance that perceived by himself. It is affected significantly by accepting or rejecting attitudes, and acceptance of positive or negative interpretations perceived by oneself or received from peers [7]. Individuals with higher self-liking accept themselves more and be calmer, comfortable, and safe in their social life. They demonstrated a higher quality of connections with their parents and peers with lower distancing, anxiety, and depression. And vice versa, lower agreeable individuals expected to find rejection and negative notes (constant criticism) from their social surroundings, decreasing their self-liking and having more problems in communicating. Furthermore, children’s self-perception as their cognitive competence and their acceptance of their peer and maternal may affect negatively and significantly their levels of being disliked by peers. Whereas, children’s physical perception is not significantly correlated with these levels [8].

Behavioral parent training (BPT) is the predominant mode of behavior management treatment targeting home-based problems for school-age youth with ADHD. BPT programs tend to cover a set of topics. Psychoeducation about ADHD and the behavioral model for treatment is often covered first. Thereafter, most BPT programs begin with teaching parents positive attending skills to improve the parent-child relationship and promote a positive family climate, as well as contingent positive consequences (e.g., praise, activity rewards, token economies/point systems) to encourage appropriate child behavior. Positive strategies are discussed first because they can interrupt the coercive cycle often displayed in families of children with ADHD [9, 10].

### The aim of the study

This study aim to compare the QOL, self-competence, and self-liking among the caregivers of ADHD and non-ADHD children.

## Methods

### Research questions

- Does self-competence of caregivers of ADHD and non-ADHD children predict being liked and disliked by themselves in a statistically significant way?
- Does self-competence of caregivers of ADHD and non-ADHD children predict their QOL in a statistically significant way?
- Does self-liking of caregivers of ADHD and non-ADHD children predict their QOL in a statistically significant way?
- Does self-competence of caregivers of ADHD and non-ADHD children predict their self-liking in a statistically significant way?

### Research design

A comparative research design was utilized in this study and was conducted between 20th of June and 21th of July 2020.

### Study setting

This study was conducted in the early intervention center for children with special needs and learning disorders in Elsinbellawin City at Dakahlia Governorate in Egypt. This center helps the children with special needs to make adaptation to their condition and helps them to learn academically and socially in contact with others in the community. The center contains 5 floors which consist of 15 rooms for children with autism, ADHD, mental retardation, and children with learning disorders; 1 room for the director; 1 room for the staff; 1 room for workers; 1 room for activity; and 1 room for play. According to the following inclusion criteria, both genders, between the ages of less than 30 and more than 40 years and free from any chronic disease, are included.

### Subjects

The samples were collected from 216 caregivers and were divided into two groups; caregivers of 108 children diagnosed to have ADHD according to DSM IV-T international criteria and with a minimum IQ ≥ 70 (investigator-estimated) were eligible for the study. General information and questionnaires were filled in by patents. The exclusion criteria comprised abnormal laboratory findings, acute or unstable medical conditions, cardiovascular disorder, history of seizures, psychosis, bipolar disorder, suicidal ideation, any medical condition that might increase sympathetic nervous system activity, or the need for psychotropic medication other than study drug. The second sample was composed of 108 normal children with the following inclusion criteria: normal children’s development not having any previous use of drugs for chronic diseases and not being treated for any psychiatric disorders. The groups were both genders. In the control group, according to caregivers, included the following criteria: agreed to contribute in the study, each of the two genders (male and female) were represented in the sample, and the mean age of the caregivers was 20–60 years.

### Sample size

Mean ± SD of QOL among ADAH group was 64.97 ± 31.92 and 76.78 ± 29.89 of control group (pilot study), and confidence level is at 95% with power of study 80%. Sample size was calculated using Open Epi and 108 caregiver for each group.

### Data collection procedure

After obtaining the required permission to conduct the study, the researchers inter-viewed the caregivers in their center in order to attain their verbal consent to participate in the study after being informed about its aim. The selected caregivers were divided into groups (5 to 10 caregivers for each group). The researchers interviewed caregivers, explained each statement to them, and then asked them to complete the questionnaire. Each group required about 30–45 min to be completed. As a result of pandemic COVID-19, the necessity of taking safety precautions such as physical distancing, wearing a mask, keeping rooms well ventilated, avoiding crowds, cleaning hands, and data collection was completed in about 1 month.

#### Data collection tool

##### Tool I: socio-demographic data and personnel characteristics

Questionnaire was established by researchers including questions about population age, gender, the level of education, marital status, job, and medical history of diseases.

##### Tool II: the WHO Quality of Life Scale-Brief (WHOQOL-Brief)

The shortened version of the original instrument WHOQOL-100, comprises of 26 questions. Two are related to an individual’s overall perception about QOL. The remaining 24 represent four domains of the original instrument; I—physical, II—psychological; III—social; and IV—environment domain [4]. The WHOQOL-Brief produces a profile with four domain scores and two individually scored items about an individual’s overall perception of quality of life and health. The four domain scores are scaled in a positive direction with higher scores indicating a higher quality of life. The study pro forma prepared in English was translated into the Arabic language (local language) and translated to Arabic to check the validity of translation by translation experts. Scoring system of QOL; low for < 50%, fair for 50–< 75%, and good for ≥ 75%.

##### Tool III: self-liking/self-competence scale

The scale was designed by [11] and consists of 16 items on 5-point Likert scale from 1 (strongly disagree) to 5 (strongly agree), of two dimensions; self-liking and self-competence. Scoring system self-competence/self-liking; low for < 50% (0–19), fair 50–< 75% (20–29), and good for ≥ 75% (30–40).

### Content validity and reliability

Validity of tools II and III were tested for their content by a jury of five experts in the field of psychiatric and mental health nursing to ascertain relevance and completeness of the tools and the needed modifications were done. Tools II and III reliability were assessed by Cronbach’s alpha through measuring their internal consistency. They show good level of reliability as follows: QOL score (α = 0.737), self-competence score (α = 0.742), and self-liking score (α = 0.741).

### Pilot study

The researchers conducted a pilot study on 10% of the study sample (20 cases, 10 from each group) to test the applicability of the data collection tool and the feasibility of the study. It was done to assess the study questionnaire clearness, easiness, and feasibility, as well as to estimate the required time to complete the questionnaire. Based on the pilot study results, some changes made to the questionnaire were mostly rephrasing and using simpler semantic for the statements, and adding some modifications was made to the instruments. Self-liking/self-competence scale (consisting of 16 items). The experts in the field of psychiatric and mental health nursing recommended classifying into two dimensions of self-esteem (8-point self-liking and 8-point self-competence). Also, define the three items need to be reversed before scoring regarding WHOQOL-Brief scale. Sample who took part in the pilot study were not included in the main study.

### Statistical data analysis

All data were collected, tabulated and statistically analyzed using SPSS 20.0 for windows (SPSS Inc., Chicago, IL, USA 2011). Quantitative data were expressed as the mean ± SD and (range), and qualitative data were expressed as absolute frequencies (number) and relative frequencies (percentage). Percent of categorical variables were compared using chi-square test. Spearman correlation coefficient was calculated to assess relationship between various study variables, (+) sign indicate direct correlation and (−) sign indicate inverse correlation, also values near to 1 indicate strong correlation and values near 0 indicate weak correlation. All tests were two sided. *P* value < 0.05 was considered statistically significant (S), and *p* value ≥ 0.05 was considered statistically insignificant (NS).

## Results

**Fig. 1:**
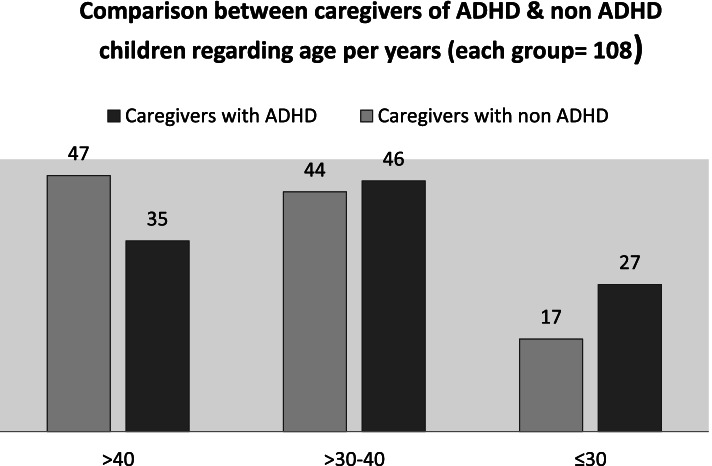
Comparison between caregivers of ADHD children and non-ADHD children regarding age per years (each group = 108)

Figure 1 represents age of caregivers of ADHD children and non-ADHD children. As for the age of caregivers of ADHD children, there are 25.00% from less than or equal to 30, and there are 42.59% that are more than 30 years old, and 32.41% who are more than 40 years old.

Comparing between caregivers of non-ADHD children and ADHD children and regarding their socio-demographic characters, Table 1 shows no statistically significant (*p* > 0.05) difference between both groups.

**Table 1:** Comparison between caregivers of non-ADHD children and ADHD children regarding socio-demographic characters (each group = 108)

Parameters	Studied groups				***χ***^**2**^	***p*** value
	Caregivers of non-ADHD children ***n*** = 108		Caregivers of ADHD children ***n*** = 108			
	No.	%	No.	%		
**Sex**						
Males	36	33.33	26	24.07	2.26	0.13
Females	72	66.67	82	75.93		
**Marital status**						
Married	70	64.81	81	75.00	2.7	0103
Single	38	35.19	27	25.00		
**Education**						
Diploma	34	31.48	45	41.67	2.9	0.24
Bachelors	59	54.63	53	49.07		
Post-graduate	15	13.89	10	9.26		
**Occupation**						
Employee	76	70.37	63	58.33	3.4	0.065
Non-working	32	29.63	45	41.67		
**Medical History of diseases**						
No	85	78.70	74	68.52	2.9	0.09
Yes	23	21.30	34	31.48		

*χ*^*2*^ chi-square test, *p* > 0.05 non-significant

Table 2 displays statistically significant difference (*p* < 0. 001) between both groups. It is apparent that all QOL dimensions of the caregivers of ADHD children were markedly deteriorated, if compared to those of non-ADHD children. The most affected QOL dimension was the psychological health (73.14%), physical health (69.45%), followed by the social relationships (66.67%), the overall QOL and general health (62.04%), and the least affected dimension was the environment dimension (60.19%). Moreover, two-third of caregivers of ADHD children suffered from poor level QOL (66.67).

**Table 2:** Comparison between caregivers of non-ADHD children and ADHD children regarding QOL dimensions (each group = 108)

	Studied groups				***χ***^**2**^	***p*** value
	Caregivers of non-ADHD children ***n*** = 108		Caregivers of ADHD children ***n*** = 108			
	No.	%	No.	%		
Overall QOL and general health						
Good	69	63.89	13	12.04	84.8	0. 001
Fair	31	28.70	28	25.92		
Poor	8	7.41	67	62.04		
Mean ± SD Range	70.6 ± 20.8 12.5–100		38.2 ± 22 0–100			
Physical health						
Good	42	38.89	4	3.70		
Fair	63	58.33	29	26.85	110.4	0. 001
Poor	3	2.78	75	69.45		
Mean ± SD Range	68.6 ± 13.7 42.86–100		38.6 ± 16.8 0–82.14			
Psychological health						
Good	18	16.67	1	0.93	93.7	0. 001
Fair	80	74.07	28	25.93		
Poor	10	9.26	79	73.14		
Mean ± SD Range	63.1 ± 11.6 41.67–83.33		42.4 ± 11.6 16.67–95.83			
Social relationships						
Good	70	64.81	8	7.41		
Fair	30	27.78	28	25.92	100.6	0. 001
Poor	8	7.41	72	66.67		
Mean ± SD Range	72.7 ± 20.1 25–100		37.1 ± 19.8 0–91.67			
*Environment*						
Good	40	37.03	8	7.41		
Fair	65	60.19	35	32.40	86.8	0. 001
Poor	3	2.78	65	60.19		
Mean ± SD Range	70.9 ± 13.9 43.75–100		45.2 ± 18.3 6.25–87.5			
WHOQOL						
Good	34	31.48	0	.00	102.7	0. 001
Fair	69	63.89	36	33.33		
Poor	5	4.63	72	66.67		
Mean ± SD Range	68.7 ± 12.4 49.04–93.27		41.3 ± 14.3 19.23–71.15			

*χ*^*2*^ chi-square test, *p* < 0.05 significant

Comparing between caregivers of non-ADHD children and ADHD children regarding self-competence and self-liking, Table 3 illustrates a statistically significant (*p* < 0.001) difference between caregivers of non-ADHD children and ADHD children. It is obvious that caregivers of non-ADHD children had good level of self-competence and self-liking.

**Table 3:** Comparison between caregivers of non-ADHD children and ADHD children regarding self-competence and self-liking (each group = 108)

	Studied groups				***χ***^**2**^	***p*** value
	Caregivers of non-ADHD children ***n*** = 108		Caregivers of ADHD children ***n*** = 108			
	No.	%	No.	%		
Self-competence						
Good	33	30.55	0	0.00		
Fair	72	66.67	91	84.26	45	0. 001
Poor	3	2.78	17	15.74		
Mean ± SD Range	27 ± 4.7 19–37		21.4 ± 4.5 9–29			
Self-liking						
Good	59	54.63	15	13.89		
Fair	49	45.37	77	71.30	48.4	0. 001
Poor	0	.00	16	14.81		
Mean ± SD Range	29.3±4.4 20-40		24.8 ± 5.4 11–36			

*χ*^2^ chi-square test, *p* < 0.05 significant

Regarding relation between QOL, self-competence, and self-liking of caregivers of non-ADHD children and their socio-demographic characters, Table 4 demonstrates that there is statistically significant relations between QOL of caregivers of non-ADHD children and their age (*p* = 0.031), sex (*p* = 0.001), marital status (*p* = 0.007), and the medical history of diseases (*p* = 0.0001). Also, that there is statistically significant relations between self-competence of caregivers of non-ADHD children and their education (*p* = 0.048), and medical history of diseases (*p* = 0.009) only. Concerning relation between self-liking of caregivers of non-ADHD children and their socio-demographic characters, there is a statistically significant relations between self-liking of caregivers of non-ADHD children and their age (*p* = 0.003), marital status (*p* = 0.002), and medical history of diseases (*p* = 0.001).

**Table 4:** Relation between QOL, self-competence, and self-liking of caregivers of non-ADHD children and their socio-demographic characters (*n* = 108)

Variables	No	QOL (***n*** = 108)						Self-competence (***n*** = 108)						Self-liking (***n*** = 108)			
		Good		Fair		Poor		Good		Fair		Poor		Good		fair	
		No	%	No	%	No	%	No	%	No	%	No	%	No	%	No	%
**Age per year**																	
≤ 30	17	9	52.94	7	41.18	1	5.88	8	47.06	9	52.94	0	.00	12	70.59	5	29.41
> 30–40	44	16	36.36	28	63.64	0	.00	16	36.36	27	61.36	1	2.27	30	68.18	14	31.82
> 40	47	9	19.15	34	72.34	4	8.51	9	19.15	36	76.60	2	4.26	17	36.17	30	63.83
*χ*^2^ (*p* value)		10.6 (0.031)						6.2 (0.18)						11.5 (0.003)			
**Sex**																	
Female	36	4	11.11	28	77.78	4	11.11	7	19.44	27	75.00	2	5.56	17	47.22	19	52.78
Male	72	30	41.67	41	56.94	1	1.39	26	36.11	45	62.50	1	1.39	42	58.33	30	41.67
*χ*^2^ (*p* value)		13.6 (0.001)						4.2 (0.12)						1.2 (0.27)			
**Marital status**																	
Married	70	28	40.00	41	58.57	1	1.43	22	31.43	47	67.14	1	1.43	46	65.71	24	34.29
Others	38	6	15.79	28	73.68	4	10.53	11	28.95	25	65.79	2	5.26	13	34.21	25	65.79
*χ*^2^ (*p* value)		9.8 (0.007)						1.4 (0. 51)						9.8 (0.002)			
**Education**																	
Diploma	34	10	29.41	24	70.59	0	.00	13	38.24	19	55.88	2	5.88	19	55.88	15	44.12
Bachelors	59	17	28.81	37	62.71	5	8.47	13	22.03	46	77.97	0	.00	31	52.54	28	47.46
Post-graduate	15	7	46.67	8	53.33	0	.00	7	46.67	7	46.67	1	6.67	9	60.00	6	40.00
*χ*^2^ (*p* value)		5.9 (0.2)						9.6 (0.048)						0.3 (0.86)			
**Occupation**																	
Employee	76	27	35.53	45	59.21	4	5.26	26	34.21	49	64.47	1	1.32	41	53.95	35	46.05
Non-working	32	7	21.88	24	75.00	1	3.13	7	21.88	23	71.88	2	6.25	18	56.25	14	43.75
*χ*^2^ (*p* value)		2.4 (0.29)						3.3 (0.19)						0.05 (0.83)			
**Medical history of diseases**																	
No	85	33	38.8	51	60.0	1	1.18	31	36.47	53	62.35	1	1.18	56	65.88	29	34.12
Yes	23	1	4.35	18	78.26	4	17.39	2	8.70	19	82.61	2	8.7	3	13.04	20	86.96
*χ*^2^ (*p* value)		18.1 (0.0001)						9.4 (0.009)						20.4 (0.0001)			

As regards the relation between QOL of caregivers of ADHD children and their socio-demographic characters, Table 5 shows that there is a statistically significant relation between QOL of caregivers of ADHD children, their occupation (*p* = 0.038), and medical history of diseases (*p* = 0.005). It seems that caregivers of ADHD children who had no work or history of medical diseases reveal fair level of QOL. As regards the relation between self-competence of caregivers of ADHD and their socio-demographic characters, there is statistically significant relation between self-competence of caregivers of ADHD children, their occupation (*p* = 0.008), and medical history of diseases (*p* = 0.008). It is obvious that the caregiver of ADHD children who works and had no history of medical diseases has a fair level of self-competence. As regards the relation between self-liking of caregivers of ADHD and their socio-demographic characters, there is a statistically significant relation between self-liking of caregivers of ADHD children, their occupation (*p* = 0.0001), and medical history of diseases (*p* = 0.003). It is evident that caregivers of ADHD children who had no work or history of medical diseases have a fair level of self-liking.

**Table 5:** Relation between QOL, self-competence, and self-liking of caregivers of ADHD children and their socio-demographic characters (*n* = 108)

Variables	No	QOL (***n*** = 108)				Self-competence (***n*** = 108)				Self-liking (***n*** = 108)					
		Fair		Poor		Fair		Poor		Good		Fair		Poor	
		No	%	No	%	No	%	No	%	No	%	No	%	No	%
**Age per year**															
≤ 30	27	14	51.85	13	48.15	23	85.19	4	14.81	6	22.22	16	59.26	5	18.52
> 30–40	46	13	28.26	33	71.74	38	82.61	8	17.39	8	17.39	32	69.57	6	13.04
> 40	35	9	25.71	26	74.29	30	85.71	5	14.29	1	2.86	29	82.86	5	14.29
*χ*^2^ (*p* value)		5.6 (0.06)				0.17 (0.92)				6.4 (0.17)					
**Sex**															
Female	26	12	46.15	14	53.8570.73	25	96.15	1	3.8519.51	4	15.38	21	80.77	1	3.85
Male	82	24	29.27	58		66	80.49	16		11	13.41	56	68.29	15	18.29
*χ*^2^ (*p* value)		2.5 (0.11)				3.6 (0.056)				1.2 (0.27)					
**Marital status**															
Married	81	25	30.86	56	69.1459.26	70	86.4277.78	11	13.5822.22	13	16.05	59	72.84	9	11.11
Others	27	11	40.74	16		21		6		2	7.41	18	66.67	7	25.93
*χ*^2^ (*p* value)		0.89 (0.35)				1.14 (0.29)				4.2 (0.12)					
**Education**															
Diploma	35	13	28.89	32	.71.11	35	77.78	10	22.22	7	15.56	28	62.22	10	22.22
Bachelors	53	19	35.85	34	64.15	46	86.79	7	13.21	8	15.10	39	73.58	6	11.32
Post-graduate	10	4	40.00	6	60.00	10	100.0	0	0.00	0	.00	10	100.00	0	.00
*χ*^2^ (*p* value)		0.75 (0.69)				9.6 (0.048)				6.8 (0.14)					
**Occupation**															
Employee	63	16	25.40	4725	74.60	5833	92.0673.33	5	7.94	3	4.76	5423	85.71	6	9.52
Non-working	45	20	44.44		55.56			12	26.67	12	26.67		51.11	10	22.22
*χ*^2^ (*p* value)		4.3 (0.038)				6.9 (0.008)				16.3(0.0001)					
**Medical history of diseases**															
No	74	31	41.89	43	58.1185.29	67	90.5470.59	7	9.46	15	20.27	52	70.27	7	9.46
Yes	34	5	14.71	29		24		10	29.41	0	.00	25	73.53	9	26.47
*χ*^2^ (*p* value)		7.7 (0.005)				7 (0.008)				11.5 (0.003)					

Table 6 shows that there is statistically significant (*p* < 0.05) correlations between QOL, self-competence, and self-liking among both caregivers of both non-ADHD children and ADHD children.

**Table 6:** Correlation between QOL, self-competence, and self-liking of caregivers of non-ADHD children and ADHD children (*n* = 108)

***Parameters***	Caregivers of non-ADHD children				Caregivers of ADHD children			
	QOL		Self-competence		QOL		Self-competence	
	(***r***)		(***r***)		(***r***)		(***r***)	
*Self-competence*	0.39	.0001*			0.58	.0001*		
*Self-liking*	0.47	0.0001*	0.67	0.0001*	0.52	0.0001*	0.41	0.0001*

*r* correlation coefficient

*Significant *p* < 0.05

## Discussion

To the best of our knowledge, this is the first study comparing the QOL, self-competence, and self-liking scores between ADHD and non-ADHD children caregivers. Although previous studies have recommended that ADHD affects both the patients and their caregivers QOL, and a larger number of investigations have pointed out that of ADHD children’s behaviors lead to increased parental stress, anxiety, and negative parenting styles which upsurge those children’s problems. Thus, we aimed to compare the impacts of ADHD on QOL, self-competence, and self-liking levels of their caregivers against those of non-ADHD children, in addition to investigation of the relationships between these personality traits. Regarding the caregivers’ socio-demographic characters, our study showed that there is no statistically significant difference between both groups of non-ADHD children and ADHD children.

By comparing QOL of the caregivers, the current study displayed a statistically significant difference between caregivers of non-ADHD children and those of ADHD children, and that two third of caregivers of ADHD children suffered from poor level QOL. As hypothesized, our results demonstrated marked impairments in all QOL dimensions in ADHD caregivers versus non-ADHD children, emphasizing how ADHD can affect the caregivers and the relevance of addressing this issue with them during treatment.

This result is matched with results found by [12] who stated that there was statistically significant difference between caregivers of non-ADHD children and those of ADHD children, and QOL of ADHD children was poor than non-ADHD children. Moreover, the most negatively affected QOL domains by ADHD in the present work were the physical and psychological health, then the social relationships of parents, in accordance with a previous study [13]. Andrade et al. [14] reported that parents of adolescents with ADHD had significantly lower QOL especially with emotional and conduct problems. In similar way, ADHD caregivers had lower QOL scores in the social relationship and environment domains when compared to the general Brazilian population (Andrade et al. 2016). Recent study mentioned that parents of children with ADHD suffer significantly higher levels of parenting stress than parents of typically developing children. They added that this highly stressed status worsens the physical and psychological QOL of the parents due to dysfunctional less effective parenting and less involvement in parent-child interaction or even child maltreatment [15]. This results are similar to [16] that indicated there were impairments in physical health, psychological health, and social interaction of caregivers of ADHD children than caregivers of non-ADHD children; this occurs due to the health condition of their children that causes exhaustion, tiredness, stress from inattention and aggressive behavior, any other symptoms of their children, and inability to make any social interaction and peer relationships with others. In the study, a statistically significant difference was found between caregivers of non-ADHD children and those of ADHD children regarding self-competence and self-liking, and that caregivers of non-ADHD children had a fair level of self-competence and good level of self-liking. This is may be because parents of ADHD children feel guilty when they cannot cope with their role demands due to exhaustion, emotional distress, family stress, and problems which reduce their efficacy. Parents of children with special needs often burden many physical or psychiatric disorders as depression due to debilitating circumstances, such as social networks isolation, loss of ability to care for children in a warm and sensitive manner, and other family conflicts [15].

The present study demonstrates that QOL of caregivers of non-ADHD children is significantly correlated with their age, sex, marital status, and their medical history of diseases. It was noticed that married female caregivers of non-ADHD children with age less than or equal 30 years who had no history of medical diseases enjoy good level of QOL. Mothers are the primary care providers for their kids due to spending most of their time with them and occupying most traditional roles, increasing the effective parenting of mothers, such as being responsive to children’s needs and being more involved in child care. When she do most of these duties, she feel happy and satisfied when she sees her children’s love and happiness. On the contrary, our present study establishes that QOL of caregivers of ADHD children is significantly correlated only with their occupation and their medical history of diseases. We detected that non-working caregivers of ADHD children who had no history of medical diseases live fair level of QOL. Work is a critical factor for parenting behaviors; as working hours, workload, and complexity increase, parents cannot fulfill the requirements of their role as both an employee and a parent, work-family conflicts appear causing psychological problems due to emotional exhaustion. Perceived satisfaction with social support availability in caregivers of children with ADHD is inversely related to parenting stress due to decrease threatening. This social support from the primary networks as families or friends to the professional networks as health care professionals, education professionals, or social services help to cope with stressful events, improving QOL [15].

In the current work, self-competence of caregivers of non-ADHD children is significantly correlated only to their education and medical history of diseases and that post-graduated caregivers of non-ADHD children who have no history of medical diseases and have a good level of self-competence, seeing that oneself is skilled, successful, and proficient in achieving goals. Seeing oneself as successful and effective in achieving goals motivates him to grow his personality and learning. Also, coping with failure and handling one’s emotions keep his development and continuous knowledge gain and education. Individuals who perceive themselves as competent and skillful of dealing with their stressful conditions or troubles are likely to take on problem-solving strategies rather than escaping strategies [15]. On the other hand, this work exhibited that self-competence of caregivers of ADHD children is significantly correlated only to their working status and medical history of diseases and that working caregivers of ADHD children who have no history of medical diseases have a fair level of self-competence.

Additionally, this study reveals that self-liking of caregivers of non-ADHD children is significantly correlated with their age, sex, marital status, and their medical history of diseases. We remarked that married female caregivers of non-ADHD children with age less than or equal 30 years who had no history of medical diseases enjoys good level of self-liking. Since the same married female caregivers of non-ADHD children with age less than or equal 30 years who had no history of medical diseases enjoys good level of both self-liking and QOL, so self-liking positively correlated with QOL, as we shall mention after. Children often told their mother that they love her and she is the best person in their world, and then she believes that she is good enough. Thus, she perceives self-worth and value, accepts herself, and be calmer, comfortable, and safe in their social life. Conversely, self-liking of caregivers of ADHD children is significantly correlated only with their working status and their medical history of diseases, similar to their self-competence. This may be attributed to marked impairments in all QOL dimensions in ADHD caregivers versus non-ADHD children due to internalizing or externalizing problems and impaired social skills or maladaptive behavior. Furthermore, we remarked that non-working caregivers of ADHD children who had no history of medical diseases reveal a fair level of self-liking beside self-competence. Non-working parents are reported to be more affected since they often experience greater feelings of guilt and responsibility for achieving their child’s needs than working parents [13]. Finally, more attention is now being paid to the quality of life of these children who live with persisting ADHD. In order for these children to achieve their full potential, interventions have been called for, like cognitive rehabilitation, physical therapy, and caregiver training.

## Conclusions

Collectively, we found that caregiver of ADHD children have fair levels of QOL, self-liking, and self-competence. We observed also that those non-ADHD children enjoy good levels of QOL, self-liking, and self-competence, emphasizing our results of presence of a statistically significant correlation between QOL, self-competence, and self-liking among both caregivers of both non-ADHD children and ADHD children. Also, these outcomes highlight the need for including the caregivers in the treatment strategies for ADHD patients to improve their QOL, self-competence, and self-liking results and promote positive outcomes.

### Limitations of the current study

Small sample sizes may limit the generalization of the results. Furthermore, we did not evaluate the impact of the child’s ADHD symptom severity on the QOL self-competence, and self-liking of their caregivers since these symptoms may affect them. Despite these limitations, our current study support that ADHD impacts the children caregivers’ QOL, self-competence, and self-liking. Therefore, a need for future studies with larger sample sizes including the impact of the child’s ADHD symptom. Also, we did not use a semi-structured interview to confirm the diagnosis, because the diagnosis and treatment are carried out inside the center by the treating doctor, and this center is specific to those children with specific needs which are internally divided into groups according to every disease and diagnosis; thus, the children of the study sample with ADHD were easily reached by the director of the center without using the semi-structured interview.

### Clinical impact

We suggest a need to increase the awareness of health professionals to identify high-risk ADHD caregivers for QOL, self-competence, and self-liking impairment. We advise also provision of design programs for families of children with ADHD in the clinical practice treatment, including family- and community-based services, besides the child behavioral training. In addition, intersectional involvement between health care system, education system, and social well-being system may decrease parents’ anxiety, depression, and sense of conflict between work and family or social isolation. In addition, intersectional involvement between health care system, education system and social wellbeing system may decrease caregivers’ anxiety, depression, and sense of conflict between work and family or social isolation, improving their QOL, self-competence, and self-liking results and promoting positive outcomes

## Data Availability

As detailed in the “References” section, all data was accessible through the Internet.

## Abbreviations

ADHD: Attention deficit hyperactive disorder
QOL: Quality of life
WHOQOL-Brief: World Health Organization Quality of Life Scale-Brief
BPT: Behavioral parent training
IQ: Intelligence quotient
DSM IV-T: Diagnostic and Statistical Manual
